# Unveiling May-Thurner Syndrome in a Case of Recurrent Deep Venous Thrombosis With Bilateral Pulmonary Embolism

**DOI:** 10.7759/cureus.63907

**Published:** 2024-07-05

**Authors:** Saviz Saghari, Olaniyi Fadeyi, Zubair Ilyas, Amirmohsen Arbabi

**Affiliations:** 1 Internal Medicine, West Anaheim Medical Center, Anaheim, USA; 2 Internal Medicine, Centinela Hospital Medical Center, Los Angeles, USA

**Keywords:** pulmonary embolism (pe), rivaroxaban, apixaban, interventional radiology, anticoagulation therapy, may-thurner syndrome, deep venous thrombosis

## Abstract

May-Thurner syndrome (MTS) is a rare cause of deep venous thrombosis (DVT), characterized by the external compression of the left common iliac vein by the right common iliac artery against bony structures. Risk factors for MTS include female sex (postpartum, multiparous, and using oral contraceptive pills), spinal abnormalities like scoliosis, prior aortoiliac vascular stent placement, dehydration, and hypercoagulability. MTS patients with partial obstruction can be asymptomatic, but progression to extensive symptomatic DVT and/or chronic venous insufficiency can occur. MTS can be diagnosed by non-invasive imaging studies including ultrasound (US), computed tomography (CT) scan, magnetic resonance imaging (MRI), venogram, catheter-based venogram, and intravascular US. For MTS patients with moderate to severe symptoms, we suggest thrombectomy, angioplasty, and stenting of the affected segment. In this case report, we highlight a 44-year-old male with a recent diagnosis of left-sided DVT on apixaban who presented with worsening leg swelling. DVT, pulmonary embolism (PE), and MTS were diagnosed with a lower extremity US, chest CT angiography, and abdominal/pelvic CT scan and venography, respectively. The patient underwent interventional radiology-guided local thrombolysis, thrombectomy, and venoplasty along with stent placement in the left common iliac vein. Subsequently, the patient was discharged on rivaroxaban.

## Introduction

May-Thurner syndrome (MTS) is characterized by the compression of the iliocaval functional unit alongside the underlying bone by the covering arterial system. Iliac vein compression between the fifth lumbar vertebra and the overlying right common iliac artery is the most common finding in MTS [1]. This anatomical variant results in high occurrence and prevalence of iliofemoral or left iliac vein thrombosis [2]. This phenomenon, initially described by May and Thurner in 1957, suggests that the chronic pulsation of the artery against the vein, coupled with compression against the lumbar vertebrae, leads to intimal hyperplasia and subsequent venous obstruction [3]. However, case studies of this condition are more frequently being reported in recent years. This can be mainly attributed to the improved imaging techniques that have allowed better visualization of iliac veins. The exact burden of MTS in the general population is still unidentified; however, there are assumptions that its prevalence might be greater than usually hypothesized [4]. Some estimates suggest that MTS accounts for 2-5% lower extremity venous disorders [5]. Patients suffering from MTS are usually asymptomatic, with most cases not being diagnosed throughout their lifetime. Females tend to have a higher incidence of MTS compared to males [6]. The pathology of MTS is attributed to the condensing of the iliac vein wall through constant irritation by the throbbing of the overlying artery. If MTS is suspected, cross-sectional imaging is necessary for its evaluation. Multi-detector computed tomography (MDCT), CT venography (CTV), and magnetic resonance venography (MRV) are effective imaging tools for detecting MTS [1]. However, catheter-based pelvic venography with intravascular ultrasound (IVUS) is the gold standard for diagnosing MTS [7,8]. These endovascular approaches are more invasive alternatives to diagnose highly suspicious cases which can be used for anticipated treatment. The management of MTS is dependent on the presence of venous thrombosis and the grade of venous stasis. Generally, short- and long-term thrombolytic medications and anticoagulants are used for the treatment of MTS. If there is a severe stenosis of the left common iliac vein, then the preferred management includes positioning of the vascular stent that resolves the compression and decreases chronic thrombotic incidents significantly [9]. In this case report, we discuss a case of a 44-year-old male presented with recurrent left lower limb deep venous thrombosis (DVT) and bilateral pulmonary embolism (PE) who was diagnosed with MTS.

## Case presentation

A 44-year-old male who works as a supervisor at a warehouse and states that he exercises regularly presented to the emergency department (ED) complaining of left leg swelling which has been worsening despite being on apixaban for a recent diagnosis of left lower extremity DVT. The patient reported that he was able to bear weight on his left leg and ambulate without issues and denies any pain in his left leg or shortness of breath. He further denies any inciting factor for DVT or past surgical history. Of note, the patient does have a family history of DVT and PE in his father who was around the same age as him. The cause of his father's DVT and PE has remained unknown with no formal diagnosis of hypercoagulable state.

**Figure 1:**
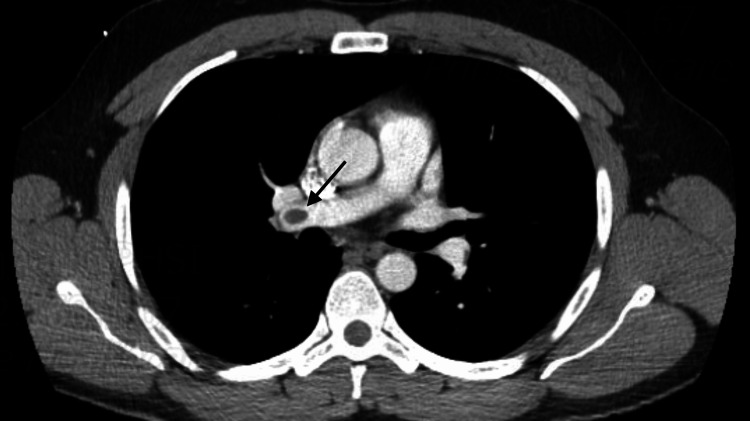
The CT angiogram of the chest with and without contrast showing pulmonary embolism CT: computed tomography

The patient was recently admitted to a nearby community hospital about one month ago and was found to have a left lower extremity DVT. He was treated with intravenous (IV) heparin during that admission, and transfer to a higher level of care facility for interventional radiology (IR) thrombectomy was considered. However, the patient showed a good response to anticoagulation, and a shared decision was made to hold off on transferring the patient at that time. Subsequently, the patient was discharged on apixaban with a plan for an initial dose of 10 mg twice daily for seven days and 5 mg twice daily thereafter and was compliant with this regimen. The patient reports that he started noticing improvement in his leg swelling the first week, but two days after switching the apixaban dose to 5 mg, his leg swelling started to worsen, and the patient presented to our ED, where a Doppler ultrasound of the left lower extremity revealed DVT involving the left common and superficial femoral veins. In the ED, the patient was started on an IV heparin drip, and the IR service was consulted with a plan for possible thrombectomy. The hematology-oncology service was also consulted for DVT with failure of anticoagulation therapy given worsening left leg DVT despite being on apixaban. Our vascular surgery service was also consulted. A CT scan of the abdomen/pelvis with and without contrast was recommended which showed a thrombus within the inferior aspect of the inferior vena cava with extension into the left common iliac vein and also into the left deep and superficial femoral veins. The CT angiography of the chest with and without contrast showed small emboli within the upper and lower lobe pulmonary artery branches bilaterally (Figure 1).

**Figure 2:**
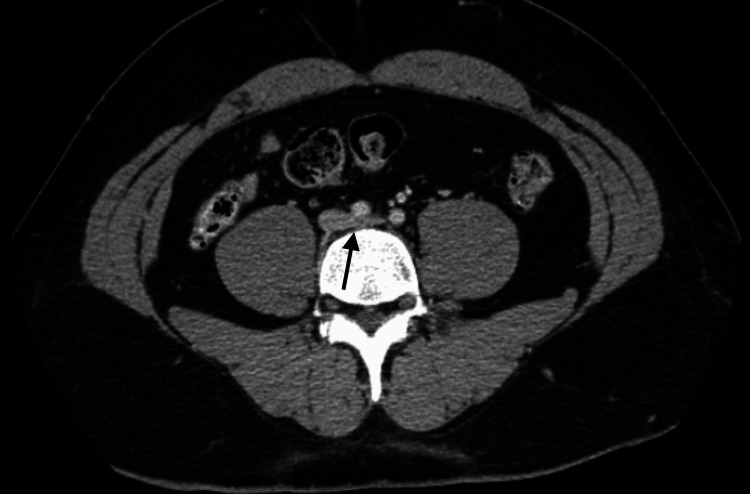
The CT angiogram of the abdomen/pelvis with and without contrast showing a slight impression upon the left iliac vein by the right iliac artery consistent with the clinical diagnosis of MTS CT: computed tomography; MTS: May-Thurner syndrome

The CT of the abdomen/pelvis with and without contrast showed a slight impression upon the left iliac vein by the right iliac artery (Figure 2) which was consistent with the diagnosis of MTS.

**Figure 3:**
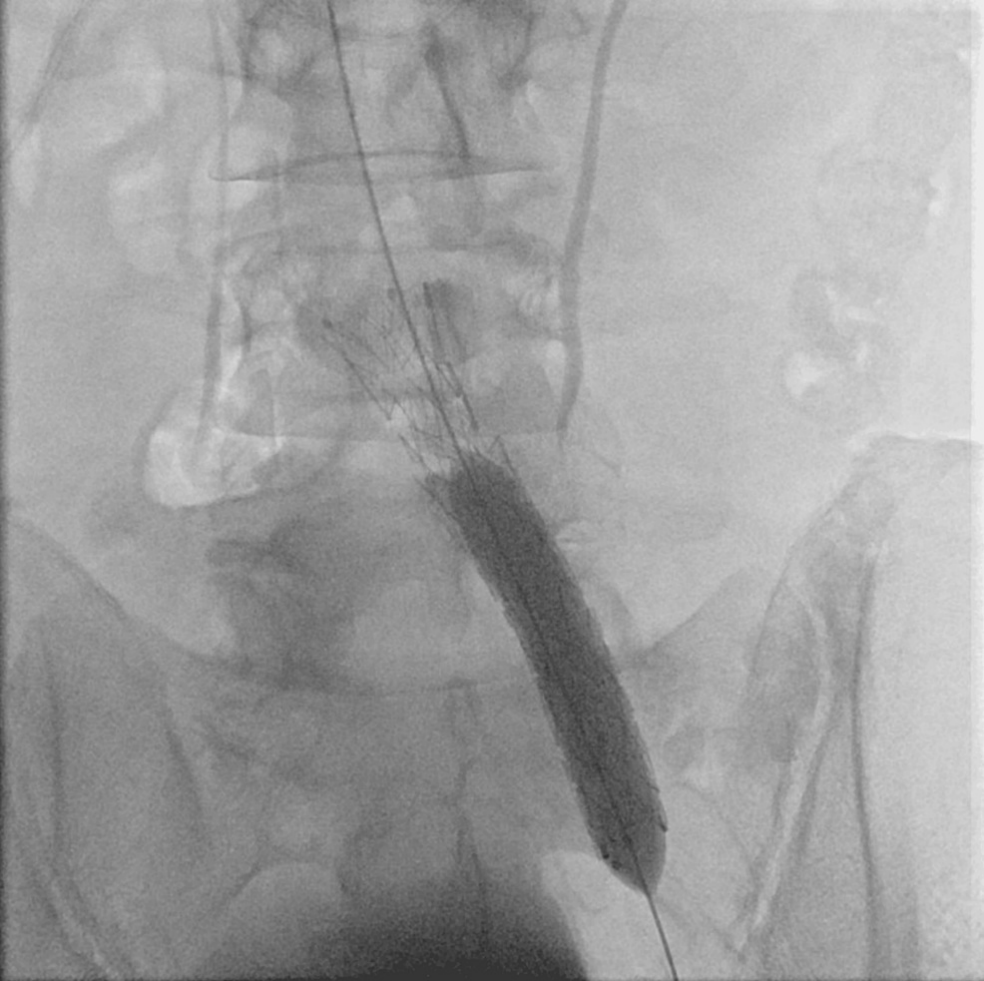
Successful completion of venogram, followed by angioplasty and stent placement in the left common iliac vein extending into the left external iliac vein

After confirmation of the MTS by imaging, the hematology-oncology specialist recommended stent placement and thrombectomy to alleviate pressure off the left iliac vein. However, the IR's initial attempt to enter through the groin was unsuccessful due to total occlusion of the left common and external iliac veins and common femoral vein along with the profunda femoris vein. The patient had to be taken back to the operation room the following day for placement of a catheter through the internal jugular vein route with recanalization of the iliac veins to get connected to the left profunda femoris vein. The procedure was followed by angioplasty with 7 mm and 10 mm balloons with a follow-up venogram that showed mural clots within the recanalized vein. A 20 cm infusion length tissue plasminogen activator (tPA) catheter was placed in the left common femoral vein extending into the left common iliac vein for overnight tPA infusion, and the patient was then transferred to the intensive care unit (ICU) for closer monitoring while on tPA drip. The following day, the patient underwent a venogram with thrombectomy and angioplasty with stent (Medtronic Abre venous self-expanding 18×100 mm stent, Dublin, Ireland) placement in the left common iliac vein extending to the left external iliac vein with restoration of the normal flow from the left common femoral vein to the inferior vena cava (Figure 3).

After being monitored in the ICU, the tPA infusion was stopped and got switched to IV heparin infusion at a rate of 18 units/kg per hour. The patient was monitored for any signs of bleeding while being on a heparin IV drip which was switched to rivaroxaban 20 mg daily upon discharge as per the hematology-oncology doctor's recommendation. The patient was discharged home with instructions to follow up with his primary care provider (PCP) and hematology-oncology doctor and a plan to continue clopidogrel 75 mg daily for one year after the date of stent placement and rivaroxaban indefinitely.

## Discussion

The present case describes a unique presentation of an MTS patient who presented with recurrent DVT and bilateral PE while being on apixaban. In the case of symptomatic MTS, continued stasis causes characteristic chronic expressions of venous hypertension including pain, thrombophlebitis, inflammation of limbs, etc. The present case described the onset of MTS occurring with swelling in the left leg. Rudolf Virchow first reported in 1851 that the higher prevalence of DVT in the left lower extremity was because of the compression of the right common iliac artery on the left common iliac vein [4]. However, the first comprehensive study was conducted by May and Thurner in 1957 in which 430 cadavers (22%) had DVT in the left lower limb [3,9].

In the present case, the CT imaging also displayed pulmonary emboli in the upper and lower lobe branches of the pulmonary artery bilaterally. These findings are in line with a recent case study by Farina et al. reporting the onset of PE in MTS patients [9]. Generally, duplex ultrasound is preferred as the first-line diagnostic modality for MTS because of its non-invasive nature and dynamical capability to assess both MTS and DVT. However, CTV can be selected if the duplex ultrasound fails to accurately evaluate the left common iliac vein (due to low sensitivity in sensing lesions situated in the superior section of the lower limb) [5]. In previous studies, CTV was considered an efficient tool for detecting the compression of the leftward common iliac vein [5,10]. However, it exposes patients to ionizing radiation, and accurately timing the venous phase contrast can be challenging leading to sub-optimal imaging results [5].

The management of MTS usually comprises two major management levels. The first level includes the prevention of progression of the DVT through anticoagulation therapy, followed by the removal of the thrombus via catheter-directed thrombectomy or thrombolysis. The second step is the modification of anatomical anomaly causing the compression of the vein often through surgical options such as bypass grafting or stenting [11]. The patient in the present case underwent a series of interventions including thrombectomy, angioplasty, and stent placement to release the pressure on the left common iliac vein. Generally, the inhibitors of factor Xa, such as apixaban and rivaroxaban, are recommended for the treatment of DVT and have shown promising results as well [12]. In our case, the initial treatment with apixaban was ineffective in avoiding the progression of the DVT, requiring more aggressive intervention with thrombectomy and stent placement. Over the years, medical treatment unaided with additional endovascular intervention has proven to be less effective [11].

The ATTRACT trial, a large multi-center randomized trial, assessed the use of anticoagulation or anticoagulation plus pharmacomechanical catheter-directed thrombolysis for the treatment of proximal (iliofemoral or femoral-popliteal) DVT [13]. The trial showed that the use of catheter-directed thrombolysis plus anticoagulation leads to a decreased incidence of post-thrombotic syndrome (PTS) over anticoagulation alone. The CAVENT trial, a prospective randomized controlled clinical trial, showed that patients with iliofemoral DVT treated with catheter-directed therapy and anticoagulation had less incidence of PTS when compared with anticoagulation alone with an absolute risk reduction of 14.4% [14].

Treatment of MTS patients with rivaroxaban has shown good results with fewer side effects. Sebastian et al., in their study, assessed the safety of rivaroxaban in patients with iliofemoral DVT, with 50% of them suffering from MTS. Their findings showed that rivaroxaban was related to lesser hazards of gastrointestinal bleeding [15]. Similar to our approach, Al Sinani et al., in their case study, also recommended anticoagulation treatment with rivaroxaban along with follow-up appointments [11]. Meng et al. also prescribed rivaroxaban (10 mg/day) to MTS patients upon discharge [16]. However, Farina et al. recommended a much higher dose rate (15 mg rivaroxaban twice a day in the first four weeks and 10 mg oral rivaroxaban with 100 mg aspirin per day for six months) compared to our study [17]. Several other case studies have also reported the use of rivaroxaban to prevent the recurrence of MTS [18,19].

## Conclusions

This case highlights severe diagnostic and managemental challenges in MTS patients. Our patient had a recurrence of the left lower extremity DVT and new-onset bilateral PE despite being on apixaban upon presentation. Further diagnostic imaging with abdominal/pelvic CT unveiled the diagnosis of MTS in this patient. The multidisciplinary approach involving IR, vascular surgery, and hematology-oncology service was crucial for managing this case. Thrombectomy, angioplasty, and stent placement effectively restored normal venous flow and alleviated the patient's symptoms. Long-term anticoagulation therapy with rivaroxaban and antiplatelet therapy with clopidogrel post-stent placement were recommended to prevent recurrence and optimize vascular health. The findings of this case study have shed light on the clinical presentation and management of MTS patients. These findings should be further explored in larger studies, with longer follow-up periods.
